# Hereditary Disease

**Published:** 1882-01

**Authors:** 


### HEREDITARY DISEASE.

There is, strictly speaking, no such thing. Children are not born diseased, however (some specific maladies excepted) much one or both parents are, but they are simply born with a predisposition to such parental malady. They are born with the material, with the powder, but actual disease will no more occur unless exciting causes are applied, than powder would detonate without the aid of fire. The observant reader has often felt surprised at seeing robust, hearty children, of parents who were seemingly at not a great remove from the grave; and if rational care were taken of such children, they would live to become healthy men and women. The practical lesson should be, a hopeful diligence in the rearing of children of diseased parentage. The difference between the children of healthy and diseased parents amounts to this: as to the latter, the powder is drier, they have less capability of resisting the causes of disease, the consequence is, a greater necessity for carefulness; this necessity is often felt, and practically attended to; the result is, that such persons are found living, scores of years after they have mouldered in the grave, who, in priding themselves on having constitutions which nothing could hurt, could not be made to feel the need of carefulness, and, consequently, perished long before their prime.

We have an instructive and royal illustration in point, in the persons of Queen Victoria and her children. Intermarriage with blood relations for ages has deeply impregnated the Guelph family with scrofula. The earlier years of the British Queen were spent in feebleness and disease, and yet she is now the apparently healthy mother of a large family of robust, healthy children, which is at once creditable to herself, and to the medical skill which dictates the hygiene of her household. The daily routine of these children is—to rise early, breakfast at eight, and dine at two. First hour after breakfast the classics; next, the modern; grammatical instruction being also carefully given; next, military exercises for the boys; then music and dancing, then the riding-school: music and drawing for the girls; then the carpenter's shop, and, occasionally, the laboratory; then shooting, and working in the royal gardens; then supper, then prayers, and then to bed.

RESULT—high bodily health, in spite of ages of "*Hereditary Tendeneies!*"

---

TRUE COURAGE—Is not so much marching up to the cannon's mouth in the hurry of battle, or mounting the scaffold for a principle, or enduring the surgeon's knife, as in living unknown and poor in a great city, striving hard, day by day, for daily bread, yet striving hopefully, resolutely, uncomplainingly and *rightfully*. Many a young heart *from the country of poor but pious parents*, comes every year to New York, and thus labors in hope of keeping dear ones at home, until life itself is worked out, and uncheered of any kindly world, unsustained by any helping hand, unaided by any pure philanthropist, unsought by any *man of God* whose mission is to seek out and *feed my lambs*, he goes down to the grave exclaiming, "*Thou, God,* ONLY *hast been my helper.*"

